# A comparison of induced and developmental cell death morphologies in lace plant (*Aponogeton madagascariensis*) leaves

**DOI:** 10.1186/s12870-014-0389-x

**Published:** 2014-12-30

**Authors:** Adrian N Dauphinee, Trevor S Warner, Arunika HLAN Gunawardena

**Affiliations:** Department of Biology, Dalhousie University, 1355 Oxford Street, Halifax, NS B3H, 4R2 Canada

**Keywords:** Programmed cell death, Vacuole, Plasma membrane, Morphology, Cell death classification, Developmental PCD, Environmentally induced PCD, Tonoplast, Live cell imaging, Autophagy

## Abstract

**Background:**

Programmed cell death (PCD) is an important process for the development and maintenance of multicellular eukaryotes. In animals, there are three morphologically distinct cell death types: apoptosis, autophagic cell death, and necrosis. The search for an all-encompassing classification system based on plant cell death morphology continues. The lace plant is a model system for studying PCD as leaf perforations form predictably via this process during development. This study induced death in cells that do not undergo developmental PCD using various degrees and types of stress (heat, salt, acid and base). Cell death was observed via live cell imaging and compared to the developmental PCD pathway.

**Results:**

Morphological similarities between developmental and induced PCD included: disappearance of anthocyanin from the vacuole, increase in vesicle formation, nuclear condensation, and fusing of vesicles containing organelles to the vacuole prior to tonoplast collapse. Plasma membrane retraction was a key feature of developmental PCD but did not occur in all induced modes of cell death.

**Conclusions:**

Regardless of the causal agent in cell death, the vacuole appeared to play a central role in dying cells. The results indicated that within a single system, various types and intensities of stress will influence cell death morphology. In order to establish a plant cell death classification system, future research should combine morphological data with biochemical and molecular data.

**Electronic supplementary material:**

The online version of this article (doi:10.1186/s12870-014-0389-x) contains supplementary material, which is available to authorized users.

## Background

Cell death processes that remove unwanted, infected, or damaged cells have evolved in eukaryotic organisms [1-3]. Traditionally, active cell death regimes have been denoted as programmed cell death (PCD), while cell death that occurs more passively has been called necrosis; however, recent studies call into question the validity of this dichotomy as there is evidence which suggests that necrosis is an active process as well [4]. PCD can be either developmentally regulated or environmentally induced [5], although significant overlap exists in the mechanisms [6]. Cell death traits differ among taxonomic groups and even histological origins within a species [3,7]. It is for this reason that efforts have been employed to create cell death classification systems, which have been primarily based on cellular morphology, and more recently, biochemical and molecular data.

In animals, there are three distinct cell death morphology types: apoptosis, autophagic cell death, and necrosis [8]. First coined by Kerr et al. [9], apoptotic morphology is characterized by a reduction of cellular volume, chromatin condensation, nuclear fragmentation, conservation of organelle ultrastructure, retention of plasma membrane (PM) integrity until an advanced stage of the death process, and subsequent formation of apoptotic bodies (Reviewed by Kroemer et al. [8]). Cells that undergo apoptosis do not cause an inflammatory reaction and are engulfed by phagocytes. Autophagic cell death is characterized by a substantial increase in autophagy prior to death [8]. Autophagic cell death in animals typically consists of an increase in autophagosomes (double membrane vesicles), which later fuse with lysosomes, and in contrast to apoptosis, there is no chromatin condensation [10]. In animals there exist three types of autophagy: microautophagy, macroautophagy, and chaperone-mediated autophagy, while in plants there are additional forms of autophagy including but not necessarily limited to mega-autophagy, involving collapse of the tonoplast, and internal degradation of chloroplasts [11]. Although autophagy, or “self-eating”, is typically a pro-survival or reparatory mechanism, it has, in certain instances, been seen to promote cell death such as in *Drosophila* salivary glands during metamorphosis (as reviewed by Green [12]). Necrosis is typically associated with cell death induced by intense stressors, and has traditionally been seen as a more passive process. Necrotic morphology has been characterized by an increase in cellular volume, organelle swelling, early PM rupture, and subsequent spilling of intracellular components [10].

Currently, there is a marked lack of consensus over the classification of different plant PCD types. In the year 2000, Fukuda placed plant PCD into three categories based on cytological features including: apoptotic-like cell death, leaf senescence, and PCD where the vacuole plays a central role [7]. According to Fukuda, the morphological hallmark for apoptotic-like cell death is a retraction of the PM from the cell wall and cytoplasmic condensation [7]. Van Doorn and Woltering in 2005 stated that no plant examples conformed to the characteristics of true apoptosis [13]. They suggest that several PCD examples appeared to be autophagic, while many other PCD types fit into neither category [13]. Reape and McCabe in 2008, and furthermore in 2013, built on the apoptotic-like cell death classification [14,15]. They discuss that despite true apoptosis not being present in plants, a number of similarities exist, specifically concerning PM retraction, which could be evolutionarily conserved [15]. Van Doorn et al., (2011) suggest there are two forms of plant PCD: vacuolar cell death and necrotic cell death, and that any use of the term apoptosis, or any derivative thereof when discussing plant PCD is a misapplication [16]. According to these authors, vacuolar cell death consists of degradation of the cell by both autophagy-like processes and the release of hydrolases immediately after tonoplast rupture [16]. Additionally, necrotic cell death is assumed to be a type of plant PCD due to the recent reports of internal signalling pathways during necrosis in animal models [16]. Alternatively, van Doorn (2011) later argued that since the vacuole is involved in almost all plant PCD types (including those not falling under the definition of vacuolar cell death), that plant PCD categories should be based on the rupture of the tonoplast in relation to cytoplasmic clearing [17]. Therefore, van Doorn [17] proposed two new categories: autolytic PCD, where rapid cytoplasmic clearing occurs post tonoplast collapse, and non-autolytic PCD, where despite the rupture of the tonoplast, no rapid cytoplasmic clearing occurs. Despite almost 15 years of attempts, well defined, workable definitions for plant PCD types based on morphology are still being developed.

**Figure 1:**
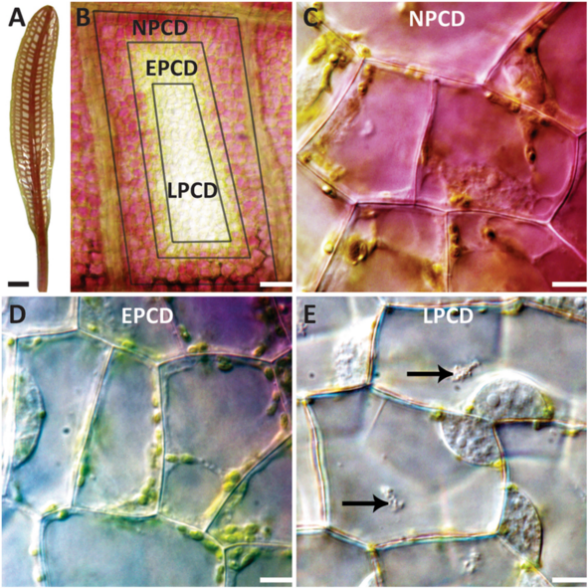
**Developmental PCD during perforation formation in the lace plant. (A)** Lace plant with window formation stage leaf. **(B)** Individual areole between longitudinal and transverse veins showing borders of all three cell types: NPCD, EPCD, and LPCD cells. **(C)** NPCD cells, pink colouration is due to anthocyanin localized to vacuoles in the underlying mesophyll cells. **(D)** EPCD cells, showing disappearance of anthocyanin but retention of chloroplasts. **(E)** LPCD cells, with few remaining chloroplasts. Large aggregates are present in the central vacuole (black arrows). Scale bars: A = 3 mm, B = 150 μm, C-E = 15 μm.

*Aponogeton madagascariensis*, also known as lace plant, is a freshwater monocot endemic to the streams of Madagascar. Lace plant leaves possess a perforated lamina and are anchored to the corm by petioles with a sheathing base [18,19]. This unique perforated leaf morphology has lent to its cultivation by aquarium enthusiasts for over 100 years [18]. Lace plant leaf perforations form via developmentally regulated PCD [20]. The lace plant is an excellent model organism for studying developmentally regulated PCD because of cell death predictability in window stage leaves (Figure 1A) between longitudinal and transverse veins (Figure 1B). Additionally, the thin, almost transparent leaves facilitate observation via live cell microscopy [5,20-22]. Within an areole, a gradient of PCD can be seen during the window stage of development, which consists of three stages: Non (NPCD; Figure 1C), early (EPCD; Figure 1D) and late PCD (LPCD; Figure 1E) [24].

PCD initiates in the centre of the areole, which is nearly void of pigment as LPCD cells have lost their anthocyanin and the majority of their chlorophyll content (Figure 1E). In LPCD cells, the vacuole swells, displacing the nucleus and cytoplasmic components towards the PM. Subsequent rupture of the tonoplast and release of hydrolytic enzymes occurs, also known as mega-autophagy. Those cells that have lost their anthocyanin, yet still have chlorophyll, are EPCD cells (Figure 1D). Interestingly, the 4-5 cell layers adjacent to the veins do not undergo PCD during leaf perforation are in the NPCD stage (Figure 1C; [23]). Unlike cells which undergo PCD, NPCD cells retain their anthocyanin throughout perforation formation (Figure 1C). After the loss of anthocyanin, changes in the chloroplasts occur, resulting in a reduction of chlorophyll. Concurrently, there is an increase in transvacuolar strands (TVS), vesicles, vacuolar aggregates, and perinuclear accumulation of both chloroplasts and mitochondria [22].

The objective of our study is to elucidate cell death morphology of different degrees and types of stressors to contribute to the formation of a plant cell death classification system. Cell death due to extreme stressors typically causes a necrotic morphology. Conversely, cell death due to mild stressors occurs more gradually and usually displays morphology typical of developmental PCD. We assume this as a hypothesis for the present work. This study utilized the unique lace plant model system to compare various environmentally induced cell death morphologies in NPCD cells to the typical developmentally regulated PCD morphology described during perforation formation using live cell imaging techniques. Recognizing how cell death morphology and the vacuole and PM in particular vary in response to different modes of induction within a single system will provide a better understanding of the intracellular dynamics of cell death.

## Results

### Developmental PCD and the framework for comparison

Leaf perforations in lace plant form via a type of developmentally regulated PCD. The morphological features that accompany this transition were defined by Wertman et al. [22] (described in the background), and were used for comparison to the induced treatments in this study (Additional file 1; Table 1). Untreated NPCD cells (which do not undergo developmental PCD during perforation formation) showed no definitive signs of cell death within a 6 h observation and were used as experimental controls to ensure that treatments triggered cell death (Additional file 2). The time for cell death to occur is represented as the mean ± standard deviation and spans from the moment a given treatment was applied until tonoplast collapse or PM retraction were observed. All videos, regardless of original acquisition times have been standardized to a length of 1 min which is responsible for the differences in playback speeds.

**Table 1:** **A comparison of lace plant cell death morphologies**

**Morphological characteristics**	**Developmental**	**Environmentally induced cell death**						
	***Leaf perforation***	***Heat***	***NaCl***		***Acid (HCl)***		***Base (NaOH)***	
		**55°C**	**400 mM**	**2 M**	**3 mM**	**12 M**	**30 mM**	**1 M**
**Anthocyanin disappearance**	**+**	**+**	**+**	**+**	**+**	**-**	**+**	**+**
**Perinuclear chloroplast formation**	**+**	**-**	**-**	**-**	**-**	**-**	**-**	**-**
**Vesicle formation**	**+**	**+**	**+**	**+**	**+**	**-**	**+**	**-**
**Vacuolar swelling**	**+**	**+**	**+**	**+**	**+**	**-**	**+**	**+**
**Nuclear condensation**	**+**	**+**	**+**	**+**	**+**	**+**	**-**	**-**
**Cessation of cytoplasmic streaming**	**+**	**+**	**+**	**+**	**+**	**+**	**+**	**+**
**Tonoplast collapse**	**+**	**+**	**+**	**+**	**+**	**+**	**+**	**+**
**PM retraction at death**	**+**	**+**	**-**	**+**	**-**	**+**	**-**	**-**
**Mean total time for death (n ≥ 3; time ± st. dev.)**	**~48 h**	**5.57 ± 0.21 h**	**5.32 ± 0.63 h**	**4.25 ± 0.38 h**	**4.02 ± 1.2 h**	**3.48 ± 1.67 min**	**33.72 ± 5.44 min**	**49 ± 5.3 s**

Morphologies of induced cell death (as observed in additional files) compared to developmental PCD during perforation formation (as delineated by Wertman et al. [22]).

Note: Time for death in the environmentally induced categories spans from the moment of treatment application until collapse of the tonoplast and PM retraction when possible.

### Heat shock experiments

**Figure 2:**
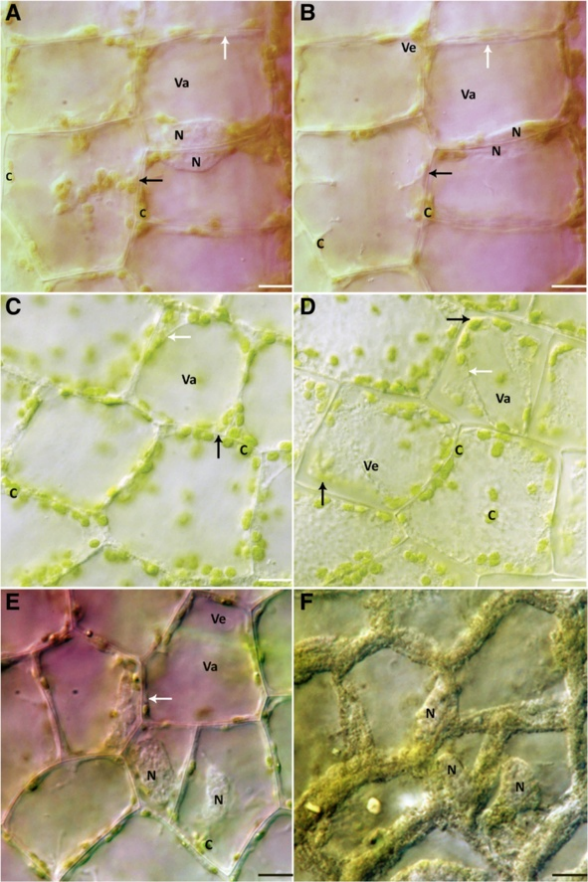
**NPCD cells after 10 min heat shock treatments. (A)** Cells at 0 h after 45°C treatment. **(B)** Cells at 6 h after 45°C treatment. Note that tonoplast and PM appears intact. **(C)** Cells at 0 h after 55°C treatment. Anthocyanin disappearance is evident. **(D)** Cells at 6 h after 55°C heat shock treatment. Vesicle formation appears throughout cells. **(E)** NPCD cells before 65°C treatment. **(F)** NPCD cells after 65°C treatment with disapearance of anthocyanin from the central vacuole. Cellular debris at the periphery of the cells had taken has a textured appearance. (A-D) N– nucleus, C– chloroplast, Va– vacuole, Ve– vesicle, white arrow– tonoplast, black arrow– PM. Scale bars: A-D = 15 μm.

Leaf pieces were treated for 10 mins at 45°C, 55°C, 65°C, and then observed microscopically. No cell death occurred within 6 h in the 45°C treatment (Figure 2A,B). The cell colouration, and the morphology of the nucleus, the chloroplasts, the vacuole, vesicles, and the PM were not observed to have changed within the 6 h (Figure 2A,B; Additional file 3). The 55°C treatment caused all cells to die in 5.57 ± 0.21 h (Figure 2C,D). Cells showed nearly slight anthocyanin disappearance shortly after the heat treatment (Figure 2C; Additional file 3). There appeared to be dramatic nuclear condensation (Additional file 4). Chloroplast abundance and shape did not appear to change (Figure 2C,D). Noticeably, there was an increase in the number of vesicles (Additional file 4). As vacuolar swelling continued, some of the vesicles appeared to fuse with the central vacuole immediately prior to tonoplast permeabilization (Additional file 4). PM retraction occurred shortly after the tonoplast collapsed (Figure 2D; Additional file 4).

Leaf pieces that were treated at 65°C had no living cells by the time they were examined under the microscope (approximately 5 mins after treatment). Therefore, images were collected before and after the heat shock treatment, respectively (Figure 2E,F). The cells exposed to this treatment underwent a change in colour from pink-purple to clearing (Figure 2E,F). The dramatic discolouration and change in the appearance of intracellular components made organelle identification difficult. Nevertheless, the comparison between images before and after the heat treatment shows a resulting granular appearance, along the margins of the cell, post-treatment (Figure 2E,F).

### Sodium chloride experiments

**Figure 3:**
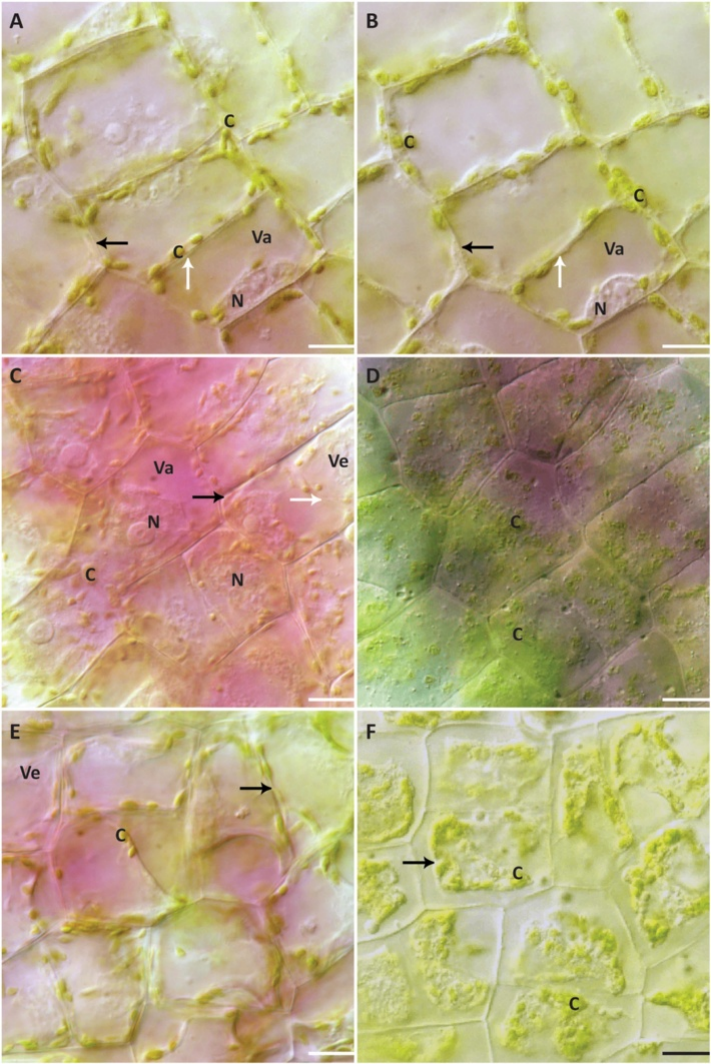
**NaCl treatment cell morphology. (A)** NPCD cells at 0 h of the 100 mM NaCl treatment. **(B)** NPCD cells at 6 h of 100 mM NaCl treatment. Chloroplasts have taken on a ‘wrinkled’ appearance. **(C)** NCPD cells at 0 h of 400 mM NaCl treatment. **(D)** Dead NPCD cells following 400 mM NaCl treatment. There is no PM retraction at cell death. **(E)** NPCD cells at 0 h of 2 M NaCl treatment. **(F)** Dead NPCD cells following 2 M NaCl treatment. Note that PM retraction occurs. (A-F) N– nucleus, C– chloroplast, Va– vacuole, Ve– vesicle, white arrow– tonoplast, black arrow– PM. Scale bars: A-F = 15 μm.

Leaf pieces were mounted in 100 mM, 400 mM and 2 M NaCl solutions and observed for 6 h (or until cell death). Leaf pieces treated with 100 mM NaCl, showed no change in cell colouration, cytoplasmic streaming, as well as nuclear and PM dynamics within the 6 h (Figure 3A,B; Additional file 5). Chloroplasts had a wrinkled appearance near the end of the observation (Additional file 5). Additionally, the size of the vacuole appeared to increase (Figure 3A,B; Additional file 4). Cells did not die within the 6 h treatment.

Leaf pieces treated with 400 mM NaCl, cell colouration appeared grey until turning greener at the moment of cell death after 5.32 ± 0.63 h (Figure 3C,D). Immediately following the start of treatment, plasmolysis occurs as evidenced by the PM peeling away from the cell wall, with filamentous structures visible between the PM and the cell wall (Additional file 6). The nucleus appeared to condense, and the chloroplasts were swollen and wrinkled later in the process (Figure 3C,D). Vesicle formation occurred in the cell, as well as a cessation of cytoplasmic streaming (Figure 3C,D). Vacuolar swelling occurred before the vesicles lysed and the tonoplast permeabilized (Figure 3C,D; Additional file 6). There was no retraction of the PM once the tonoplast collapsed (Additional file 6). The cell colouration that changed from pink to green occurred while the vesicles lysed (Additional file 6).

For leaf pieces treated with 2 M NaCl, cell colouration changed gradually and eventually became green as cell death progressed over a 4.25 ± 0.38 h timeframe (Figure 3E,F). After application of the treatment, the PM peeled away from the cell wall. Some filamentous structures connecting the PM to the cell wall were observed, but very few in comparison to the 400 mM NaCl treatment. There was very little cytoplasmic movement, and later in the cell death some vesicles were observed. Vacuolar swelling and tonoplast rupture were observed. Late in the death process, numerous spherical opaque bodies of various sizes were prominent and could be seen merging together (Figure 3E,F; Additional file 7). As the cells died, these spherical bodies shrank and disappeared as PM retraction occurred (Additional file 7).

### Acid and base experiments

**Figure 4:**
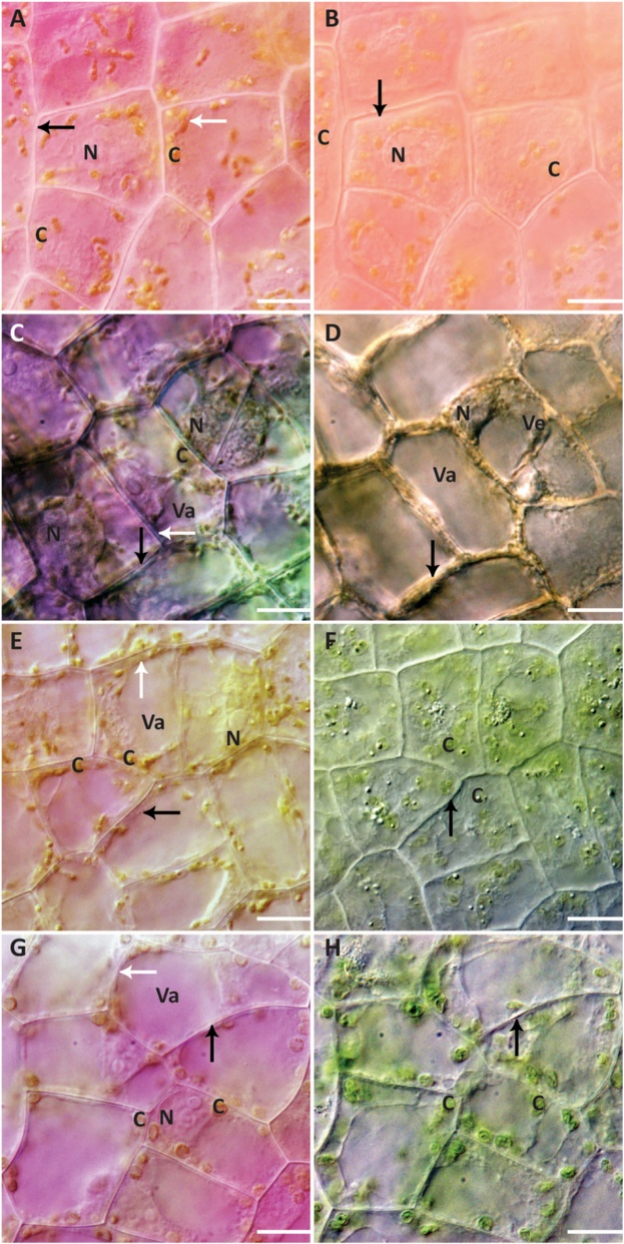
**Acid and alkaline treatment cell morphology. (A)** NPCD cells at 0 h for the 12 M HCl treatment. **(B)** NPCD cells at cell death after the 12 M HCl treatment. Nuclear condensation was evident. PM retraction was observed. Anthocyanin colouration remains, although a slight colour change occurs. **(C)** NPCD cells at 0 h for the 3 mM HCl treatment. **(D)** NPCD cells after death induced by 3 mM HCl. Anthocyanin disappearance occurs throughout treatment. **(E)** NPCD cells at 0 h for the 30 mM NaOH treatment. **(F)** NPCD cells at cell death for the 30 mM NaOH treatment. No retraction of the PM occurs. Note the swollen and ‘wrinkled’ appearance of the chloroplasts. **(G)** NPCD cells at 0 h for the 1 M NaOH treatment. **(H)** NPCD cells after death for the 1 M NaOH treatment. No PM retraction occurs. Note the swollen and ‘wrinkled’ appearance of the chloroplasts. **(A-H)** N– nucleus, C– chloroplast, Va– vacuole, Ve– vesicle, white arrow– tonoplast, black arrow– PM. Scale bars: A-H = 25 μm.

The acid and base concentrations chosen for the experiments represent the most severe that were feasible (12 M HCl, 1 M NaOH), and those least severe but still triggering cell death within 6 h (3 mM HCl, 30 mM NaOH). Leaf pieces were treated with 12 M HCl, 3 mM HCl, 30 mM NaOH, and 1 M NaOH solutions and observed as mentioned above. In the 12 M HCl solution, the mean time for cell death was 3.48 ± 1.67 min. The cell colouration immediately changed, with cells appearing bright pink (Additional file 8). Immediately before nuclear condensation, the nucleoli appeared to swell (Additional file 8). Nuclear, chloroplast, and vacuole condensation appeared to occur simultaneously (Additional file 8). No differences in vesicle formation were observed. Tonoplast collapse occurred before retraction of the PM from the cell (Figure 4A,B; Additional file 8).

The cells of leaf pieces treated with 3 mM HCl solution died 4.02 ± 1.02 h following treatment. Increasing translucency of the cell accompanied by loss of colour was observed (Figure 4C,D; Additional file 9). Swelling of the vacuole occurred concurrently with vesicle formation, and the condensation of the nucleus and chloroplasts occurred immediately before cell death (Additional file 9). There was an evident increase in aggregate number and size, in addition to an increase in TVS throughout the treatment (Additional file 9). In previous studies, these aggregates were found to contain organelles such as chloroplasts and mitochondria [22]. Tonoplast collapse was evident in the later stages of death and the PM did not retract (Figure 4C,D; Additional file 9).

For leaf pieces treated with a 30 mM NaOH solution, the mean time for cell death was 33.72 ± 5.44 min. Treatment led to a change in cell colouration from pink to blue/green prior to clearing (Figure 4E,F; Additional file 10). Nuclear displacement occurred progressively throughout the treatment (Additional file 10). Throughout the death process, there was an increase in the number of vesicles (Additional file 10). At the time of cellular death, chloroplasts appeared swollen (Figure 4F). The size of the vesicles appeared to be larger than those seen in other induced treatments (Additional file 10). In tandem with the increase in vesiculation was an apparent increase in the size of the vacuole (Additional file 10). Collapse of the tonoplast was immediately preceded by either collapse of vesicle membranes, or fusion with the central vacuole (Additional file 10). Inside both the vesicles and the vacuole, precipitates appeared immediately before collapse (Additional file 10). Simultaneous with the tonoplast collapse was dramatic swelling of the chloroplasts (Additional file 10). There was no retraction of the PM, however, it was not believed to have remained intact (Figure 4F; Additional file 10). Treatment of lace plant leaf sheath tissue with 30 mM NaOH showed the same cell death features as reported for NPCD cells, but also allowed for a clearer view of vesicles within the cell compared to leaf tissue (Additional file 11). Aggregates, and intracellular components, including whole organelles like chloroplasts, were observed to enter the vesicles and fuse with the central vacuole immediately prior to cell death (Additional file 11).

For leaf pieces treated with a 1 M NaOH solution, the mean time for induced cell death was 49 ± 5.3 s. Cell colouration changed from pink to blue and then to green post cell death (Additional file 12). The nucleus disappeared immediately before cell death (Figure 4G,H; Additional file 12). Chloroplasts swelled either immediately before or during vesicular and vacuolar collapse (Figure 4H; Additional file 12). Vesicular collapse either preceded or occurred in tandem with vacuolar collapse (Additional file 12). The PM did not retract and appeared to lose its integrity (Figure 4G,H; Additional file 12).

## Discussion

Heat shock treatments have been found to alter cell metabolism, disrupt mitochondria, and result in an increase in ROS [23,25]. Depending on the severity, heat shock will result in cell death. In the 55°C treatment, anthocyanin disappearance was apparent immediately after treatment. It is suspected by the authors, that a spike in ROS may have played a role in anthocyanin disappearance, although anthocyanin is also a heat sensitive pigment [26]. In addition to anthocyanin disappearance, there was a marked increase in the number of vesicles within the cell. The characteristic increase in vesicles, the appearance of organelles in the vacuole, and an increased volume of the central vacuole, up until tonoplast collapse, provides evidence for both macro-, and mega-autophagy. The retraction of the PM from the cell wall after tonoplast collapse resembles the PM retraction observed during lace plant developmental PCD, however the ultrastructure of the PM at this point was not investigated. Although, it should be noted that the cell corpse in developmental PCD exhibits a more condensed morphology in comparison. Similarly, PM shrinkage has been shown in heat shock experiments using lace plant protoplasts [23]. Surprisingly, the protoplasts were less susceptible to heat shock at 55°C than the *in situ* cells in our experiment, with the protoplasts undergoing PCD after 20 min while the *in situ* cells underwent PCD after 10 min. The severity of the 65°C treatment resulted in cell death before completion of the treatment. The 65°C cell death morphology appeared remarkably different compared to the 55°C treatment, lacking PM retraction and with a loss of chlorophyll from the chloroplasts. The textured appearance along the periphery of the cell is believed to be the remains of cellular debris. Membranes within the cell are not believed to have retained their integrity. The subsequent morphology of the 65°C treatment is characteristic of what is commonly considered necrotic cell death [15,16].

In the 100 mM NaCl treatment, there was a dramatic slowing in cytoplasmic streaming. Sodium chloride stress has been implicated in an increase in cytoplasmic Ca^2+^, which can arrest cytoplasmic streaming, by Na^+^ displacing Ca^2+^ from the PM, and from liberating Ca^2+^ from internal stores [27]. However, there is little research that assesses the effects of salinity on cytoplasmic streaming [28]. In the 100 mM and 400 mM NaCl treatments, the chloroplasts took on a wrinkled appearance. This wrinkled effect on chloroplast ultrastructure has similarly been observed in TEM images of tomato cells grown in a medium containing 100 mM NaCl [29]. Chloroplasts appeared swollen in the 400 mM NaCl treatment, but this effect was not observed in the 2 M NaCl experiments. In potato cultivars, electron microscopy showed that although the structural integrity of cells appeared intact, the chloroplasts appeared swollen when plants were irrigated with 100 mM, and 200 mM NaCl solution, respectively [30]. Swollen chloroplasts have also been seen in wheat and sweet potato leaves under salt stress [30,31].

Interestingly, the vacuole appeared to increase in size in the 100 mM NaCl treatment, which occurs similarly in LPCD cells during leaf perforation developmental PCD. This rapid increase in vacuole size, in response to saline conditions, has been demonstrated in suspension-cultured of mangrove cells and barley root meristematic cells [32]. Na^+^ accumulation in the central vacuole and subsequent increase in vacuolar volumes has been shown to be an active process and is believed to be one strategy employed by the cell in response to salt stress [32]. Vesicle formation occurred in the 400 mM and 2 M NaCl treatments, suggesting an increase in macro-autophagy, perhaps to recycle damaged intracellular components [33]. High salt solutions have been shown to elicit autophagy in *Arabidopsis thaliana* by up-regulating autophagy related genes [33].The initial retraction of the PM from the cell wall in the 400 mM and 2 M NaCl treatments, in contrast to the late-stage PM retraction seen in developmental PCD, is plasmolysis and is due to changes in osmotic pressure. The filamentous structures between the PM and the cell wall observed during plasmolysis are speculated to be hechtian strands ([34]; Additional files 6, and 7). Interestingly, there were many more of these strands in the 400 mM NaCal compared to the 2 M NaCl treatment. At the final stages of death there was a contrast between these two treatments; cell treated with 400 mM NaCl exhibited tonoplast rupture and no PM retraction, whereas the 2 M NaCl treatment had a significant PM retraction. The authors speculate that the numerous strands connecting to the cell wall in the 400 mM NaCl treatment group played a role in negating the PM retraction which occurred after treatment at the higher concentration.

The most striking characteristic of the 12 M HCl treatment is the surprising dramatic retraction of the PM from the cell wall. The PM retraction in the 12 M HCl treatment resembled PM retraction seen at the end of developmentally regulated PCD in lace plant perforation formation. Although this retraction appears to be morphologically similar, this cell death lies in contrast to PCD in perforation formation, which typically takes several days. The cell death process in the 12 M HCl treatment was rapid and although the ultrastructural changes to the PM are unknown, the authors suspect this is a passive process. In the 3 mM HCl treatment, cytoplasmic streaming slowed, which may have been a result of either a change in cytoplasmic pH or an increase in cytosolic Ca^2+^. The vacuole swelled extensively, which may have been a cellular response to extracellular acidity by increasing the volume of the vacuole, as it is generally more acidic than the cytoplasm under normal conditions. The swelling of the vacuole in this treatment resembled swelling in LPCD cells during developmental PCD. The observation of vesicles was similar to the salt stress treatments, and may be indicative of an increase in macro-autophagy. Cell death occurred with the permeabilization of the tonoplast. In the 30 mM NaOH treatment, there was a considerable increase in vesicles compared to other treatments, which may also indicate an increase in macro-autophagy. The change in colour of the vacuole from pink to blue/green immediately before cell death suggests that the vacuolar pH was dramatically raised to near alkalinity as anthocyanin’s visible colour shifts.

Comparison between induced cell death and its developmental counterpart revealed that there are several common characteristics, including cessation of cytoplasmic streaming and tonoplast collapse (Table 1). Vacuolar dynamics appear to be consistent among the developmental and induced cell death videos (Table 1), and occupying the majority of a plant cell, it is likely to make a substantial contribution to cell death processes. Perinuclear chloroplast formations only occurred during developmental cell death. Likewise, the 12 M HCl treatment was the only cell death without anthocyanin disappearance, which is likely due to the response of the pigment to the low pH of the solution. In the NaOH treatments, nuclear condensation was not observed, in contrast to all other cell death types. Vesicle formation was a common characteristic of all cell death types except those that resulted in very rapid cell death, such as the 12 M HCL and 1 M NaOH treatments. PM retraction was seen in perforation formation, the 55°C, 2 M NaCl and 12 M HCl treatment groups. Interestingly, in all cases where cell death was observed, the vacuole played a central role, specifically seen with tonoplast collapse occurring in all cell death types. The results of this comparative study are summarized in Table 1.

In animal cells, there exists a morphologic classification system of cell death types, with three categories: apoptosis, autophagic cell death, and necrosis. Among the most apopotic-like characteristic seen in the lace plant is retraction of the PM due to a reduction in cell volume observed during leaf perforation developmental PCD. A similar morphology can be seen in the 55°C treatment, 2 M NaCl and the 12 M HCl treatment. Regarding autophagic cell death, an increase in vacuolar swelling and vesicle formation was observed in heat, salt, and most pH treatments. Notably, an increase in vesicles was not observed in the most extreme pH treatments (12 M HCl, 1 M NaOH). In most cell death morphologies induced by less severe stressors, such as in 30 mM NaOH treatment in leaf sheath tissue, whole organelles encapsulated by a vesicle were seen to fuse with the vacuole prior to tonoplast collapse. Recently, a dual role of autophagy as either an initiator of PCD during the HR (hypersensitive response), or a downstream executioner during developmental PCD has been proposed by Minina et al. [36]. The current authors believe that the examples of autophagy shown here are acting downstream, perhaps through the activation of some components similar to the lace plant leaf perforation developmental pathway. Interestingly, the high number of spherical opaque bodies which formed in the 2 M NaCl treatment fused with others that were in close proximity, and either disappeared or shrunk before PM retraction and cell death, but more research is needed to determine their composition and function.

Necrotic features such as early rupture of the PM, were typically seen in the most extreme treatments. A reduction of cellular volume, along with the active retraction of the PM is typically associated with a slower, more internally regulated form of cell death; however, PM retraction was seen in the most extreme acid treatment (12 M HCl) and took place within minutes. While the PM retractions observed in the 55°C and 2 M NaCl treatments were morphologically similar to the 12 M HCl treatment, the timeframe for cell death to occur was much longer in comparison. Considering the relatively slow timeframe for cell death from the 55°C and 2 M NaCl treatments (5.57 ± 0.21 h and 4.25 ± 0.38 h, respectively), the authors hypothesize that the PM retraction is an active process, whereas the 12 M HCl treatment represents a necrotic collapse. Further investigation is required, however, to determine whether or not the various induced cell death morphologies shown here are forms of PCD.

In 2000, Fukuda proposed the existence of three PCD categories in plants: apoptotic-like, leaf senescence, and one in which the vacuole plays a central role [7]. Since then, there have been several proposed classification systems for plant PCD, but currently none are unanimously accepted. Proposed classifications have often centered on characteristics of a particular organelle, notably the vacuole, or PM. The PM is commonly used due to its conspicuous appearance when retracted from the cell wall. This retraction, along with a reduction of cellular volume are characteristics present in apoptosis but absent in necrosis in animal models. Emphasis on the vacuole is likely due to its expansive nature in plant cells, often occupying up to 90 % of cellular volume which is unlike any autolytic organelle found in animal cells. Also, the vacuole is known for its autolytic properties, participating in cellular processes such as autophagy, which are associated to cell death events. Macro-autophagy occurs when cellular components are sequestered to the vacuole via double membrane vesicles. Vacuolar swelling followed by tonoplast collapse, known as mega-autophagy, is common in plant cell death processes, as was observed in this study. It is very possible that future cell death classifications will center on the role of the vacuole in plant cell death, as has been seen in previous classification proposals. Our data indicates that individual stressors typically result in different cell death morphologies amongst differing stressor intensities, despite being within a single system. Other researchers are encouraged to consider the means by which induced PCD studies are carried out. Although the experiments in this paper used isolated stressors, it has been found that more damage occurs to plants when multiple stressors occur simultaneously [35], and therefore it may be considered that treatments should replicate combined stressors that would occur naturally.

## Conclusions

Lace plant (*A. madagascariensis*) is an ideal model organism for studying both developmental PCD and induced cell death, specifically due to its thin, near-transparent leaves which are suitable for live-cell microscopy. Using live-cell imaging, similarities and dissimilarities were demarcated among the induced treatments and developmental lace plant PCD. Cell death was successfully induced with physical and chemical stressors including heat shock, NaCl, HCl and NaOH. Although there were significant differences among the treatments, the vacuole played a central role as mega-autophagy was present in all forms of cell death that were observed. This study illuminates the variability of cell death morphology using different stressors and the authors believe that morphological data, though important should, be coupled with biochemical and molecular data before it can significantly contribute toward the formation of a cell death classification system in plants.

## Methods

### Aquarium grown plants

*Aponogeton madagascariensis* plants were grown in three aquariums containing freshwater which was supplemented on a weekly basis with: 1 mg/L monopotassium phosphate, 10 mg/L potassium nitrate and 3 mg/L CSM + B plantex (Aquarium Fertilizers, Napa, California, USA). Aquarium light intensity was approximately 125 μmol m^-2^ s^-1^, which was produced from an aqua glow T8 fluorescent light bulb (Hagen, Montréal, Québec, Canada). The aquariums were maintained on a 12 h light/dark cycle and kept in a room maintained at 24°C.

### Tissue preparation, experimental design, & control treatment

Experimental lace plant tissue was derived from window stage leaves and utilized NPCD cells, which would not undergo developmental PCD in perforation formation. Leaves were removed from the plant at the petiole and rinsed in dH_2_O. The midrib was excised and the subsequent tissue was sectioned into approximate 5 mm^2^ pieces. Before treatments, tissue pieces were maintained in dH_2_O with a 6.7-7.0 pH range at ambient temperature. All tissue was taken from a leaf the same day as the experiment. A tissue piece was placed on a microscope slide and the appropriate solution was added to the slide and then covered and sealed with VALAP (paraffin wax, lanolin, petroleum jelly mixture in a 2:1:1 ratio). There was a minimum of three independent replicates for each experiment with tissue derived from different leaves. Experiments ran for 6 h or until cell death occurred, which was determined by either tonoplast collapse or PM retraction. For all treatments, various levels of intensity for each stressor were optimized. All treatments presented here were also tested on whole leaves using the custom slide method of Wertman et al. [22] to ensure that the same cell death morphologies occurred in a whole organ and so comparisons could be drawn to the previously described lace plant developmental PCD pathway. This study utilized leaf sections to obtain higher resolution videos and due to logistical constraints when applying the extreme acid and base treatments at the microscope, which was necessary as cell death occurred rapidly. Controls were mounted in dH_2_O, at ambient temperature. The solution was first placed on the mounted tissue to create a wet mount and then the cover slide was sealed with VALAP.

### Heat treatments

A water bath (VWR International, Radnor, Pennsylvania, USA) was set to the desired temperature, and filled with water. A beaker filled with dH_2_O was placed into the centre of the water bath. Once the set temperature (45°C, 55°C, or 65°C) was attained, a piece of tissue was submerged within the beaker. After 10 min, the tissue was removed, mounted on a slide in dH_2_O (at ambient temperature) to create a wet mount, and then sealed with a cover slip with VALAP.

### Sodium chloride treatments

Solutions of 100 mM, 400 mM and 2 M NaCl (>99.5% Purity; SIGMA-ALDRICH Inc., St. Louis, Missouri, USA) were prepared. Tissue was mounted on a slide, the appropriate solution was added to create a wet mount, and the cover glass was sealed on all sides with VALAP.

### Acid and base treatments

For solutions of moderate pH (3 mM HCl, 30 mM NaOH), the solution was added to the slide before placing and then sealing the cover slip. For solutions that were strongly acidic or basic, respectively, (12 M HCl, 1 M NaOH), the cover slip was sealed on two opposing sides, and the solution was added once the slide was mounted on the microscope. Observation ceased once cells were determined to be dead (no more than 6 h). A video using leaf sheath tissue with the 30 mM NaOH treatment was used to provide a clearer image of the vesicles.

### Light microscopy, video and image acquisition, editing and time analysis

Videos and images of epidermal, NPCD stage cells, cells which do not undergo developmental PCD, were taken using DIC optics on a Nikon 90i (Nikon Canada, Mississauga, Ontario, Canada) microscope fitted with a DXM 1200c digital camera. Data acquisition was made using NIS Elements AR 3.10 software (Nikon Canada, Mississauga, Ontario, Canada). Digital photographs were taken with a Nikon Coolpix L110 camera (Nikon Canada, Mississauga, Ontario, Canada). Image edits were made with Adobe Photoshop and Adobe Illustrator (Adobe Systems Inc., San Jose, California, USA). Video editing was made with Adobe Premier Pro CS5 (Adobe Systems Inc., San Jose, California, USA). All additional file videos were standardized to 1 min duration regardless of original duration length and this standardization accounts for differing playback speeds. The time for cell death to occur is represented as the mean ± standard deviation and spans from the moment a given treatment was applied until tonoplast collapse or PM retraction were observed. Data are represented as the mean ± standard deviation.

## Additional files

Additional file 1:
**Developmental PCD gradient within a single areole over 30 min.** NPCD stage cells (right) do not undergo PCD during leaf morphogenesis and contain anthocyanin pigmentation in the mesophyll. Cells within the EPCD and LPCD stages do not contain anthocyanin. Several of the nuclei are visible, including their nucleoli. Note the reduction in the number of chloroplasts in LPCD cells in comparison with EPCD cells. Large aggregates form in EPCD cells and are readily apparent in LPCD cells. TVS increase notably in EPCD cells but are less frequent in LPCD cells. Approximately 30 playback speed. Additional file 2:
**Control NPCD cells.** Untreated NPCD cells mounted in distilled water over a 6 h observation period. The cells show no obvious signs of stress and do not die within a 6 h period and thus serve as a control for comparison with the induced cell death treatments. Approximately 360× playback speed. Additional file 3:
**NPCD cells treated at 45°C for 10 min.** Cells in this heat shock treatment largely resemble untreated NPCD cells. Nuclear displacement may be due to changes in vacuolar dimensions. TVS are visible and are denoted. No cell death occurs. Approximately 360× playback speed. Additional file 4:
**NPCD cells treated at 55°C for 10 min.** Anthocyanin disappeared from the underlying mesophyll cells during the heat shock process. Increase in vesicle formation along periphery of cells occurs throughout the treatment. Tonoplast collapse and PM retraction from the cell wall occur simultaneously. Approximately 360× playback speed. Additional file 5:
**NPCD cells treated with 100 mM NaCl solution.** Large aggregates form in the central vacuole of the cells. TVS increase throughout the treatment. Chloroplast appearance changes throughout treatment to appear ‘wrinkled’. The PM appears to remain intact throughout treatment and no retraction occurs. No cell death occurs. Approximately 360× playback speed. Additional file 6:
**NPCD cells treated with 400 mM NaCl solution.** Hechtian strands present between PM and cell wall. Note the plasmolyzed cells at the beginning. Chloroplasts appear to swell throughout the treatment while appearing ‘wrinkled’. Tonoplast collapse and PM retraction occurs at cell death. Approximately 300× playback speed. Additional file 7:
**NPCD cells treated with 2 M NaCl solution.** Plasmolysis occurs rapidly after contact with the solution. Hechtian strands are present between the PM and cell wall, but are not abundant. Note the spherical bodies that form before cell death. Throughout the process, anthocyanin pigmentation in the tissue slowly changes colour and becomes green as the cells die. Swelling occurs prior to tonoplast collapse and a dramatic retraction of the PM. Approximately 300× playback speed. Additional file 8:
**NPCD cell treated with 12 M HCl solution.** Cell death occurs extremely quickly due to the severity of the stressor. The pink colour from vacuolar anthocyanin does not disappear at the time of cell death in the presence of the low pH solution. Notable is the momentary swelling of the nucleoli immediately before nuclear condensation. Tonoplast collapse and PM retraction from the cell wall occur simultaneously. Approximately 1.25× playback speed. Additional file 9:
**NCPD cells treated with 3 mM HCl solution.** Note nuclear displacement may be due to changes in vacuolar dimensions. A slowdown of cytoplasmic streaming occurs and later there an increase in vesicle formation immediately before tonoplast collapse. Tonoplast collapse and nuclear condensation occur simultaneously. No PM retraction from the cell wall occurs. Approximately 180× playback speed. Additional file 10:
**NPCD cells treated with 30 mM NaOH solution.** An increase in vesicle formation occurs and they fuse with the vacuole before tonoplast collapse. Immediately prior to tonoplast collapse, a large amount of precipitates in the vacuole are visible. Note the swollen appearance of the chloroplasts. No PM retraction occurs. Approximately 45× playback speed. Additional file 11:
**Leaf sheath tissue treated with 30 mM NaOH solution.** Following exposure to 30 mM NaOH there is an abundance of vesicles within cells. Chloroplasts are visible within vesicles, which fuse with the central vacuole immediately prior to tonoplast collapse. No PM retraction is observed. Approximately 25× playback speed. Additional file 12:
**NPCD cells treated with 1 M NaOH solution.** An increased in vesicle formation occurs, and later the vesicles fuse to the central vacuole immediately before tonoplast collapse. Prior to cell death there is a dramatic change in colour due to the effect of NaOH on anthocyanin in the underlying mesophyll. Also, nuclei disappeared and large amounts of precipitates are present in the vacuole immediately before tonoplast collapse. Note the swollen appearance of the chloroplasts. No PM retraction occurs. Approximately 2.5× playback speed.

## Abbreviations

EPCD: Early programmed cell death
LPCD: Late programmed cell death
NPCD: Non-programmed cell death
PCD: Programmed cell death
PM: Plasma membrane
ROS: Reactive oxygen species
TVS: Transvacuolar strands
